# Posterior Cortical Atrophy: Does Complaint Match the Impairment? A Neuropsychological and FDG-PET Study

**DOI:** 10.3389/fneur.2019.01010

**Published:** 2019-09-20

**Authors:** Laura Guerrier, Camille Cransac, Bérengère Pages, Laure Saint-Aubert, Pierre Payoux, Patrice Péran, Jérémie Pariente

**Affiliations:** ^1^ToNIC, Toulouse NeuroImaging Centre, University of Toulouse, Inserm, UPS, Toulouse, France; ^2^Department of Neurology, University Hospital of Toulouse, Toulouse, France; ^3^Department of Nuclear Medicine, University Hospital of Toulouse, Toulouse, France

**Keywords:** posterior cortical atrophy, Alzheimer's disease, FDG-positron emission tomography, neuropsychological assessment, visuospatial and visuoperceptive dysfunction, patient's complaint

## Abstract

**Objective:** Posterior Cortical Atrophy (PCA) is a neurodegenerative disease characterized predominantly by visual impairment. However, diagnosis of PCA remains complicated with an interval of several years between initial reporting of symptoms and diagnosis. The aim of the present study is to define if patients' visual and gestural complaints are consistent with their clinical profile.

**Method:** An evaluation of daily visual problems as well as a full neuropsychological assessment and FDG-PET were performed in 15 PCA patients. We compared glucose metabolism between these PCA patients and 18 healthy controls. Correlation analyses were conducted in PCA patients between visual and gestural complaint, clinical impairments, and brain glucose metabolism.

**Results:** Major impairment of cognitive functions was detected in PCA patients specifically in visual domains. Positive correlations were found between visual impairments and hypometabolism in the right temporo-parieto-occipital cortices. However, no correlation was found between complaint and visual impairment in PCA patients.

**Discussion:** Our main results suggest a consistent relationship between clinical impairment and brain metabolism. However, the patient's complaint and visual performance are not linked. Combining the literature and our results, it seems that patients are generally aware of difficulties but misinterpret them. This misinterpretation may be responsible for the delayed diagnosis.

## Introduction

Posterior cortical atrophy (PCA) is characterized by an insidious onset, gradual progression and prominent early disturbance of visual functions (1). Currently, PCA is considered as an atypical form of Alzheimer's disease (AD) (2, 3) with biomarkers consistent with Alzheimer's pathophysiology in 80% of PCA cases (4). However, PCA diagnosis remains complex and may take several years after first complaint by the patient (5). As the first symptoms are visual, patients are initially referred to an ophthalmologist. Ophthalmological examination is normal in this condition. Neurological expertise is subsequently required, sometimes years after the first symptoms (6). One hypothesis to explain this delay could be a misunderstanding of their impairments by the patients themselves. Although insight seems to be preserved in PCA patients (1), this aspect has never been clearly investigated and literature of PCA clinical cases remains controversial (2, 7). In order to identify patients' complaints more clearly and facilitate the diagnosis for ophthalmologists and neurologists, Croisile and Mollion (6) proposed the PCA questionnaire (PCA-Q) to evaluate visual and gestural complaints (6). The authors reported significantly more visual and gestural complaints in PCA patients than in typical AD patients and healthy controls. However, the relationship between complaint and gnosis and praxis in PCA patients has not yet been established.

Two clinical variants of PCA including ventral and dorsal PCA are described in the literature. Visuospatial functions, impairment, apraxia, agraphia, features of Balint syndrome (optic ataxia, simultagnosia, ocular apraxia), or Gerstman syndrome (digital agnosia, acalculia) are characteristic of dorsal PCA variant. Impairment of visuoperceptive functions, visual agnosia, and prosopagnosia are characteristic of the ventral PCA variant (8).

Clinical symptoms have been linked to cortical atrophy and hypometabolism in dorsal (occipito-parietal) and ventral (occipito-temporal) regions (9, 10). To explore the link between visual deficits and cortical thickness measured using MRI in 21 PCA patients, Lehmann et al. (11) reported a lower cortical thickness in occipito-temporal and occipito-parietal areas in relation to visuoperceptual and visuospatial deficits, respectively (11). PCA is also characterized by major hypometabolism in occipital, parietal associative as well as temporo-parietal cortices (4, 12, 13). In a study using positron emission tomography (PET) and [^18^F]fluorodeoxyglucose (FDG), Nestor et al. (14) reported a change in metabolism in occipito-parietal regions in PCA patients presenting with predominant visuospatial deficits compared to healthy controls. Asymmetry indicative of more pronounced hypometabolism in the right hemisphere was also reported (14).

The main objective of this study was to understand whether patient complaint reliably reflects cognitive impairment in PCA patients. We also explored the link between complaint and brain glucose metabolism since literature suggests that a decrease in brain glucose metabolism explains cognitive impairment in PCA (11, 14). An important first step was to confirm the existence of such a link between clinical impairments and brain glucose metabolism in our population. We assessed visual and gestural complaint by the Croisile and Mollion questionnaire (6), clinical impairment by a comprehensive neuropsychological evaluation and brain metabolism using FDG-PET in 15 PCA patients.

## Methods

Fifteen PCA patients were recruited through the outpatient Memory Clinic of The Neurology Department of Toulouse University Hospital (France). The clinical diagnosis of PCA was carried out in accordance with the clinical criteria provided by Crutch et al. (4) and was retrospectively confirmed by the revised 2017 criteria (8): progressive decline in visual abilities, relatively intact memory, and language functions in the early stages and atrophy of posterior regions of the brain (4). All visual deficits were explained by cognitive and non-ophthalmic impairments. Cerebrospinal fluid (CSF) samples were obtained by lumbar puncture from all patients. CSF levels of total Tau (T-Tau), phospho Tau (p-Tau), Aβ42, and Aβ40 were measured. Ratios were also calculated from these biomarkers, including the Innotest Amyloid Tau Index [IATI = Aβ42/(240 + (1.18 × T-Tau)] and the Aβ42/Aβ40 ratio (15). PCA patients were deemed to have AD pathophysiology if they met the following criteria: phospho-Tau ≥60 pg/mL and Innotest Amyloid Tau Index ≤ 0.8 or Aβ42/Aβ40 <0.045 (16).

For the purpose of imaging analysis, 18 healthy controls (HC) matched in age, gender and education were enrolled in this study. The HC had no history or clinical evidence of psychological or neurological disorders and were not cognitively impaired.

This study was approved by the local ethics committee (Comité de Protection des Personnes Sud-Ouest et Outre-Mer I) and the French Agency for Safety and Security of Medical Devices (Agence Française de Sécurité Sanitaire des Produits de Santé, reference A90605-58).

### Visual and Gestural Complaint Assessment

The PCA-Q was completed by each PCA patient (6). The PCA-Q comprises 32 questions regarding daily visual and gestural difficulties (see French and English versions of the questionnaire in Supplementary Figures 1A,B, respectively). This questionnaire covers visual and gestural domains and comprises 32 items, sub-divided into 12 sections. For each item, a score of 1 is allocated if a complaint is reported and a score of 0 in the absence of any complaint. A visual complaint sub-score (/15) and a gestural complaint sub-score (/9) were computed based on this questionnaire. The items that constitute each sub-score are detailed in Appendix A1 (Supplementary Material).

### Assessment of Visual Functions

PCA patients completed an assessment of visual and gestural functions. Due to important impairments in these domains, some tests could not be completed by all patients (interrupted after the example phase). Only tests used for the statistical analysis are described below (for the complete description and results of visual and gestural assessment (see Appendix A2 in Supplementary Material and Supplementary Table 1, respectively).

Visual functions were assessed using tests from the Visual Object and Space Perception battery (VOSP) battery (17, 18). Primary visual capacities were assessed with the *Shape Detection Screening* test. Visual perceptive functioning was assessed with the 2 following tests: the *Silhouettes* test, assessing the capacity to identify objects depicted from unusual perspectives, and the *Object Decision* test, assessing the capacity to select the silhouette drawing of a real object among three silhouettes of non-sense objects. Visual spatial functions were evaluated with the *Dot counting* test to identify the number of stimuli presented in random array and the *Position Discrimination* test to discriminate relative spatial position. Each visual test started with an example phase. For each test, patients who failed at the example phase were considered untestable. Tests with more than 5 untestable patients (representing 1/3 of patients) were removed from the analyses (see details in Appendix A2 in Supplementary Material).

To address the problem of missing data due to untestable patients and in agreement with the previous study (11), raw scores were transformed into rank scores. Patients with a missing score were assigned the score of the lowest performance of the group minus 1 point. Thus, patients who did not pass the example phase are given a lower score than those who completed the test. Raw scores were then converted to rank scores among the 15 PCA patients, ranging from 1 for the lowest performance to 15 for the highest performance. A visual performance score (/75) was computed from the rank sum (/15) of the five following tests: *Shape Detection, Dot Counting, Position Discrimination, Silhouettes*, and *Object Decision*.

### Assessment of Gestural Functions

Gestural functions were evaluated by Mahieux's test (19) with right and left *symbolic gestures* and *pantomimes*. A gestural performance score (/20) was derived from the sum of the raw scores in the following Mahieux's tests: *symbolic gestures to the right (/5)* and *left (/5)*, and *pantomimes (/10)*.

### Other Neuropsychological Assessment

Patients underwent the Mini-Mental Scale Examination (MMSE) (20) to assess global cognitive functions, and the Free and Cued Selective Reminding Test (FCSRT) (21) to assess verbal memory. The Digit Span Forward and Backward test [Weschler Adult Intelligence scale III—WAIS III, (22)] was used to assess verbal working memory. Phonemic and semantic fluency (23) were used to assess initiation and the Similarities test [WAIS-IV, (24)] for abstract verbal conceptualization. A dictation task (writing of 5 regular and 5 irregular words and 3 sentences) and a reading task (8 regular words, 8 irregular words and 8 pseudo-words) were used to assess agraphia and alexia, respectively.

### Brain Imaging

#### Data Acquisition

All 18 HC and 15 PCA patients underwent an [^18^F]FDG-PET scan. Acquisitions were performed on a Biograph 6 TruePoint Hirez (Siemens Medical Solutions, Munich, Germany) hybrid PET/computed tomography (CT) scanner (3D detection mode, producing images with 1 × 1 × 1.5 mm voxels). Cerebral emission scans began around 20 min after the injection of 1.85 MBq/kg weight of [^18^F]FDG, and lasted 10 min. A structural T1-weighted image was also recorded for each participant.

#### Data Processing and Analyses

The pre-processing of [^18^F]FDG-PET data was performed using Statistical Parametric Mapping software (SPM12; Wellcome Trust Centre for Neuroimaging, London, UK) running on MATLAB (Version 2016b, MathWorks, Inc.). For each participant, Standardized Uptake Value (SUV) images corrected for body weight, the rescale slope and the injected dose (corrected by the interval between injection time and scan time) (25) were created. Rigid co-registration onto the corresponding T1-weighted image was then applied. The partial volume effect using the T1-weighted image was corrected with the PET-PVE12 toolbox (26) running on MATLAB. Spatial normalization of [^18^F]FDG PET images in the MNI space was achieved using the [^18^F]FDG PET template developed by Della Rosa et al. (27). Normalized images were then scaled quantitatively using the vermis of the cerebellum as the reference region to obtain the SUV ratio (SUVR). Finally, SUVR images were smoothed with an 8 mm full width at half maximum (FWHM) Gaussian Kernel.

### Statistical Analysis

#### Demographic and Neuropsychological Data

All statistical analyses on demographics as well as on cognitive, visual and gestural performance were performed using the R project package, version 3.5.1. A threshold of *p* < 0.05 was used for significance.

Intergroup comparisons on age and level of education were performed using Student's *t*-test for independent samples and on sex with Chi-Square test.

In order to investigate the relationship between complaint and deficits, Spearman correlations were investigated between the PCA-Q visual complaint sub-score and the visual performance score, and between the PCA-Q gestural complaint sub-score and the gestural performance score.

#### Brain Imaging Data

Intergroup comparisons using voxel-based analysis (voxel level, *p* < 0.05, FWE-corrected, cluster > 50) were performed for [^18^F]FDG-PET imaging. In order to investigate metabolism asymmetry, median SUVR were extracted for the following 7 left and right regions of interest (28) from the DTK (Desikan-Killiany-Tourville) atlas (29): frontal, lateral temporal, medial temporal, lateral parietal, medial parietal, and occipital cortices. Details of these ROIs are reported in Supplementary Table 2. A hemispheric asymmetry index (AI) was then calculated for each ROI using the formula AI [%] = −200 × (R – L)/(R + L) (30). Positive scores indicate more pronounced hypometabolism in the right hemisphere while negative scores indicate more pronounced hypometabolism in the left hemisphere. The difference in AI between healthy subjects and PCA patients was assessed using a Wilcoxon-Mann-Whitney signed-ranked test.

Voxel wise correlations were performed in PCA patients between [^18^F]FDG uptake and the following variables (voxel level *p* < 0.001, cluster > 50 voxels): visual performance score (/75), gestural performance score (/20), PCA-Q visual sub-scores and PCA-Q gestural sub-score. In order to investigate more precisely the relationship between metabolism and visuoperceptive and visuospatial functions, voxel-wise correlations were also conducted between [^18^F]FDG uptake and each of the 5 variables constituting the visual performance score (voxel level *p* < 0.001, cluster > 50 voxels). Correlations using regions of interest (ROI) were also established between the median SUVR of each ROI (left and right) and the afore-mentioned variables.

## Results

PCA patients and HC were comparable in age (PCA: 66 [10], HC: 68 [6], *p* = 0.38), level of education in years (PCA: 12 [1], HC 11.5 [7], *p* = 0.898), and sex (PCA: 7 females/8 males, HC: 12 females/6 males, *p* = 0.435). Among the 15 patients, 12 had CSF biomarkers consistent with amyloid pathology. Three patients presented with normal levels of amyloid and tau protein (Table 1).

**Table 1:** Demographic data and neuropsychological performance for each PCA patient.

**Patient ID**	**1**	**2**	**3**	**4**	**5**	**6**	**7**	**8**	**9**	**10**	**11**	**12**	**13**	**14**	**15**	**PCA Median [IQR]**	**N under 5th %ile (%)**
AD biomarker profile (CSF)	+	+	+	+	+	+	–	+	+	+	+	–	+	–	+		
Tau (pg/mL)	148	348	173	424	340	933	275	1,179	659	531	166	156	584	149	1,373	348 [452]	
Phospho-Tau (pg/mL)	29	58	36	62	62	109	58	178	81	64	30	53	104	43	218	60 [37]	
Aβ42 (pg/mL)	208	365	435	308	373	361	217	287	149	190	96	526	331	1,074	284	414 [352]	
Aβ40 (pg/mL)	5,839		14,877	5,671			4,627				2,196	4,718		6,560			
Diagnostic delay (years)	10	4	1	13	1	1	4	3	2	3	2	5	0.5	0.5	5	3 [3.57]	
**Demographic data**																	
Age	68	70	74	66	64	67	66	59	77	58	53	66	69	53	58	66 [10]	
Gender	M	F	M	F	F	F	F	M	M	F	M	F	M	F	F	–	
Socio-cultural level (years)	11	5	10	12	16	11	11	12	17	17	12	12	12	11	12	12 [1]	
**General functions**																	
MMSE /30	17	20	24	9	15	14	28	22	25	20	15	15	23	25	12	20 [8.5]	
**PCA-Q**																	
Total /32	15	10	25	26	27	8	18	17	19	12	10	27	9	14	19	17 [11]	
Visual questions /15	6	6	10	11	14	4	7	6	11	6	6	12	7	5	9	7 [4.5]	
Gestural questions /9	5	2	7	7	5	2	6	5	4	3	1	8	0	3	8	5 [4]	
**Primary visual capacities**																	
Shape detection /20	8	14	17	UT	UT	16	15	17	19	17	UT	UT	20	14	13	16 [3]	9 (60%)
**Visual perceptive functions**																	
Silhouettes /30	UT	7	17	UT	UT	6	8	13	14	7	2	UT	11	27	UT	9.5 [6.8]	13 (86.7%)
Object Decision /20	UT	8	11	UT	UT	6	11	18	11	7	8	UT	13	15	UT	11 [4.5]	13 (86.7%)
**Visual spatial functions**																	
Dot counting /10	2	2	7	UT	UT	4	8	5	7	9	0	UT	10	10	6	6.5 [4.8]	13 (86.7%)
Position discrimination /20	11	10	17	UT	UT	15	16	16	15	15	UT	UT	18	13	UT	15 [2.5]	15 (100%)
Visual performance score (sum of rank, /75)	23.5	36	61.5	13.5	13.5	40	54.5	59.5	58.5	50.5	24	13.5	68.5	59	24		
**Gestural functions**																	
Symbolic gestures, right /5	4	5	5	0	4	3	5	5	5	5	3	5	5	4	2	5 [1.5]	4 (26.6%)
Symbolic gestures, left /5	4	5	5	0	1	3	5	5	5	5	2	2	5	4	2	4 [3]	6 (40%)
Pantomimes /10	9	5	9	1	7	10	10	10	10	9	4	9	10	10	6	9 [3.5]	5 (33.3%)
Gestural performance score (sum of raw scores, /20)	17	15	19	1	12	16	20	20	20	19	9	16	20	18	10		

*Raw scores for each patient are presented, along with the median and interquartile group range. The number and percentage of PCA patients with scores below the 5th percentile are reported where possible. A positive AD CSF biomarker profile corresponds to: phospho-Tau ≥60 pg/mL and Innotest Amyloid Tau Index ≤ 0.8 or Aβ42/Aβ40 <0.045. PCA, Posterior Cortical Atrophy; AD, Alzheimer's Disease; IQR, Inter-quartile Range; +, positive, –, negative; CSF, Cerebrospinal Fluid; MMSE, Mini-Mental State Examination; PCA-Q, Posterior Cortical Atrophy Questionnaire; VOSP, Visual Object and Space Perception Battery; UT, Untestable*.

All patients reported a cognitive complaint at the PCA-Q with a minimal complaint of 8/32 and a median value of 17/32.

Details on the neuropsychological assessment are reported in Supplementary Table 3. With regard to the 5 visual tests, more than half of the patients systematically performed below the 5th percentile (between 60 and 100%). For gestural domains, between 26.6 and 40% of the patients performed below the 5th percentile.

**Figure 1:**
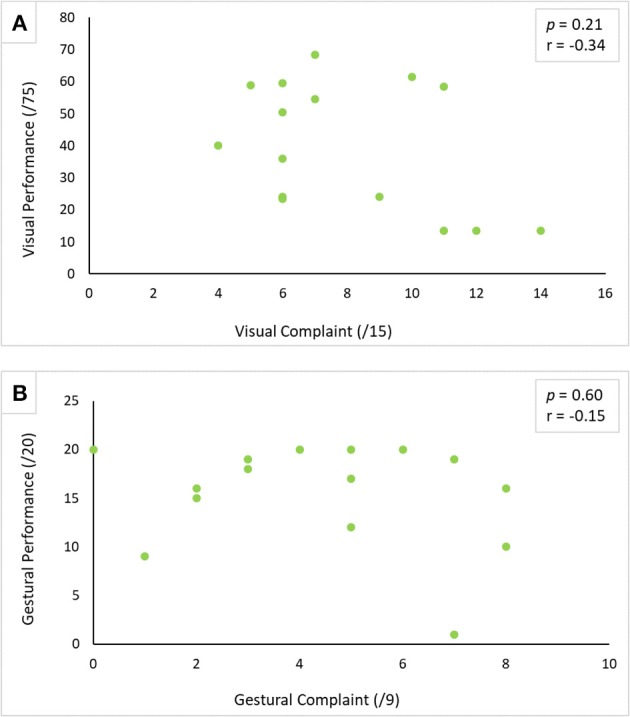
Correlation between performance and complaint in visual **(A)** and gestural **(B)** modality. The correlation was carried out using Spearman's correlation test with a threshold of *p* < 0.05.

Similarly, no correlation was established between visual complaint and visual performance (r = −0.34, *p* = 0.21) (see Figure 1A), and between gestural complaint and gestural performance (r = −0.15, *p* = 0.60) (see Figure 1B).

**Figure 2:**
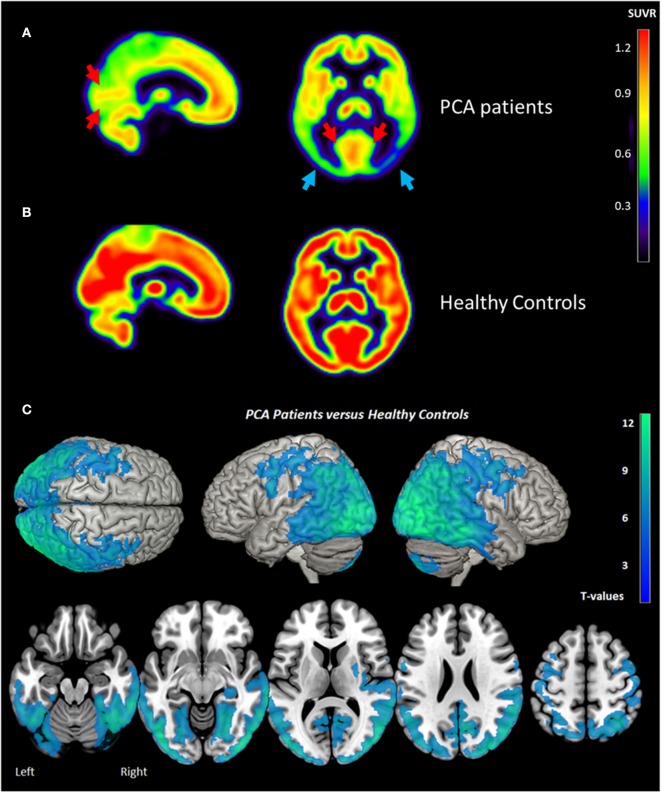
Brain metabolism in PCA Patients **(A)**, Healthy Controls **(B)**, and comparison between PCA patients and Healthy Controls **(C)** measured by [18F] FDG PET. “Occipital tunnel sign” is represented by preserved uptake in the medial occipital areas (red arrows) in comparison to lateral occipital (blue arrows). Metabolism asymmetry with right predominance (blue arrows) is also found in PCA patients. **(A,B)** represent the mean of PCA patients (*n* = 15) and healthy controls (*n* = 18), respectively. The statistical threshold for group comparison is pFWE-corr < 0.05 (k > 50 voxels). PCA, posterior cortical atrophy; SUVR, Standard Uptake Value Ratio.

**Figure 3:**
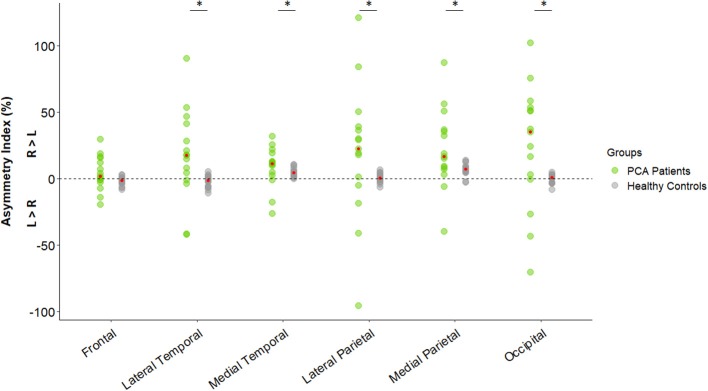
Hemispheric asymmetry index of [18F]FDG uptake in regions of interest in healthy subjects (*n* = 18) and PCA patients (*n* = 15). Dot plot representing asymmetry index [%] using the formula: −200 × (R – L)/(R + L). Red dots represent the median for each group. Differences between healthy controls and PCA patients were assessed using the Wilcoxon-Mann-Whitney signed-ranked test. *p < 0.05. R, Right; L, Left; PCA, Posterior Cortical Atrophy.

Hypometabolism was detected in PCA patients compared to healthy subjects in the temporo-parieto-occipital cortices, frontal areas (bilateral frontal eye field) and cerebellum (vermis excluded) (see Figure 2C). More severe hypometabolism was found in the lateral occipital compared to the medial occipital cortex in PCA patients (see Figures 2A,B), a signature referred to as “occipital tunnel sign.” Hypometabolism was predominant in the right hemisphere, with significantly lower right uptake in the medial and lateral temporal cortex, the medial and lateral parietal cortex, and occipital cortex (see Figures 2A, 3). Details on FDG uptake in each hemisphere as well as AI are reported in the Supplementary Table 4.

**Figure 4:**
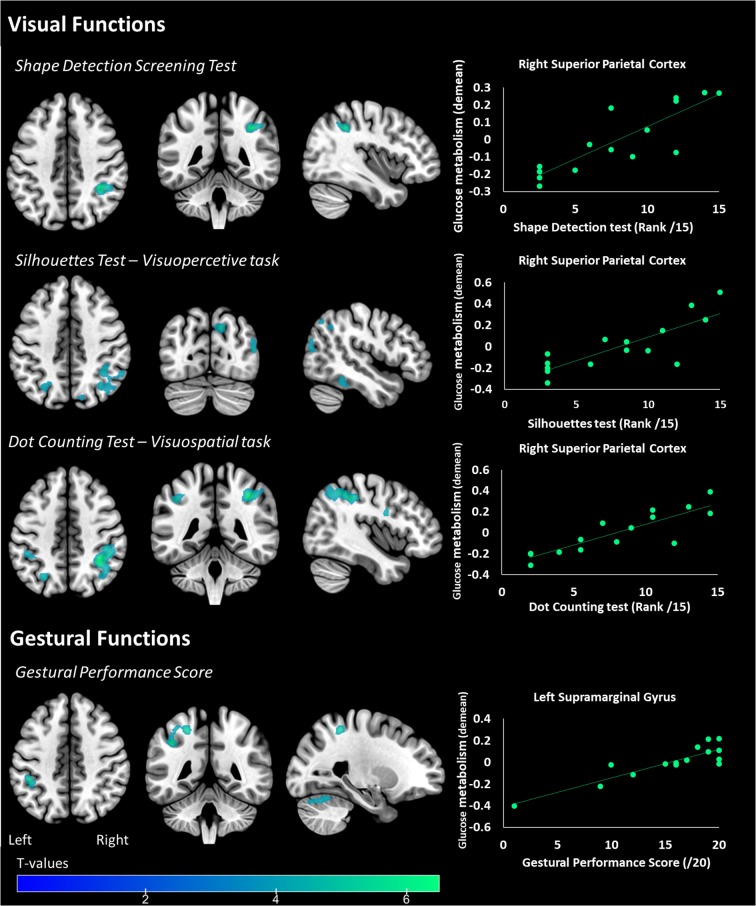
Areas of the brain where metabolism correlates with primary visual functions (Shape detection Screening Test), visuoperceptive (Silhouettes Test), visuospatial (Dot Counting Test), and gestural functions in PCA patients. The statistical threshold is puncorr < 0.001 (k > 50 voxels). Correlations on the right panel are performed on the whole cluster considered (i.e., the right superior parietal cortex for visual functions and the left supramarginal gyrus for gestural functions).

Regarding visual functions, performance in the *Shape Detection, Silhouettes* and *Dot Counting* tests was positively correlated to [^18^F]FDG uptake in the right parieto-occipital cortex using the voxel-wise approach (see Table 2 and Figure 4). Similar results were recorded using the ROI approach (see Table 3). In addition, a positive correlation was found between performance in the *Object Decision* test and [^18^F]FDG uptake in the right lateral parietal cortex using the ROI approach, but not using a voxel-wise approach (see Table 3). No correlation was found between performance in the *Position Discrimination* test and [^18^F]FDG uptake, regardless of the approach used.

**Table 2:** Areas of the brain where metabolism correlates with visual and gestural functions in PCA patients.

	**Anatomical structures**	**Number of voxels**	**MNI coordinates**			**T**
			**x**	**y**	**z**	
**VISUAL FUNCTIONS**						
**Visual performance score (/75)**						
	Right superior parietal cortex	165	36	−44	42	6.11
	Right precuneus	59	24	−60	22	4.7
**Shape detection screening test**						
	Right superior parietal cortex	190	36	−44	40	6.97
	Right precuneus	80	24	−58	24	4.66
	Right superior parietal cortex	50	24	−52	60	4.52
**Silhouettes test—visual perceptive task**						
	Right precuneus	132	12	−74	38	5.73
	Right superior parietal cortex	373	36	−48	42	4.79
	Right middle occipital gyrus	97	48	−76	16	4.75
	Left superior parietal cortex	104	−24	−66	48	4.67
	Right parietal operculum	99	36	−28	20	4.65
	Right inferior temporal gyrus	73	48	−42	20	4.64
**Dot counting test—visual spatial task**						
	Right superior parietal cortex	801	36	−50	46	6.88
	Left supramarginal gyrus	114	−46	−40	42	6.34
	Left superior parietal cortex	85	−26	−66	46	5.08
	Right precentral gyrus	54	44	0	26	5.03
	Left middle occipital gyrus	66	−38	−86	32	4.95
	Right precuneus	68	26	−58	16	4.82
	Right superior occipital gyrus	53	30	−76	26	4.19
**GESTURAL FUNCTIONS**						
**Gestural performance score (/20)**						
	Left supramarginal gyrus	343	−42	−44	38	6.4
	Cerebellum, left lobule VI	312	−22	−74	−24	4.62

**Table 3:** Correlation matrix between metabolism and visual functions, gestural functions, visual complaint, and gestural complaint.

	**Left frontal cortex**	**Right frontal cortex**	**Left lateral temporal cortex**	**Right lateral temporal cortex**	**Left medial temporal cortex**	**Right medial temporal cortex**	**Left lateral parietal cortex**	**Right lateral parietal cortex**	**Left medial parietal cortex**	**Right medial parietal cortex**	**Left occipital cortex**	**Right occipital cortex**
**VISUAL FUNCTIONS**												
Visual performance score (/75)	−0.34 (NS)	−0.14 (NS)	−0.01 (NS)	0.06 (NS)	−0.28 (NS)	−0.32 (NS)	**0.55 (*****p*** **=** **0.032)**	**0.65 (*****p*** **=** **0.008)**	0.49 (NS)	**0.61 (*****p*** **=** **0.016)**	0.34 (NS)	0.45 (NS)
**Primary visual functions**												
Shape detection	−0.45 (NS)	−0.22 (NS)	−0.22 (NS)	−0.09 (NS)	−0.39 (NS)	−0.4 (NS)	0.41 (NS)	**0.56 (*****p*** **=** **0.029)**	0.36 (NS)	**0.52 (*****p*** **=** **0.045)**	0.16 (NS)	0.28 (NS)
**Visual perceptive functions**												
Silhouettes	−0.2 (NS)	−0.02 (NS)	0.18 (NS)	0.26 (NS)	−0.15 (NS)	−0.12 (NS)	**0.64 (*****p*** **=** **0.011)**	**0.74 (*****p*** **=** **0.002)**	**0.54 (*****p*** **=** **0.039)**	**0.67 (*****p*** **=** **0.007)**	0.41 (NS)	**0.53 (*****p*** **=** **0.042)**
Object decision	−0.28 (NS)	−0.14 (NS)	0.12 (NS)	0.14 (NS)	−0.17 (NS)	−0.20 (NS)	0.47 (NS)	**0.53 (*****p*** **=** **0.044)**	0.39 (NS)	0.48 (NS)	0.32 (NS)	0.44 (NS)
**Visual spatial functions**												
Dot counting	−0.35 (NS)	−0.16 (NS)	0.01 (NS)	0.02 (NS)	−0.25 (NS)	−0.36 (NS)	**0.64 (*****p*** **=** **0.01)**	**0.69 (*****p*** **=** **0.0042)**	**0.6 (*****p*** **=** **0.019)**	**0.62 (*****p*** **=** **0.013)**	**0.54 (*****p*** **=** **0.036)**	**0.56 (*****p*** **=** **0.029)**
Position discrimination	−0.27 (NS)	−0.09 (NS)	−0.14 (NS)	−0.06 (NS)	−0.33 (NS)	−0.36 (NS)	0.35 (NS)	0.45 (NS)	0.34 (NS)	0.47 (NS)	0.09 (NS)	0.23 (NS)
**GESTURAL FUNCTIONS**												
Gestural performance score (/20)	−0.03 (NS)	−0.14 (NS)	0.19 (NS)	−0.22 (NS)	0.06 (NS)	−0.37 (NS)	**0.7 (*****p*** **=** **0.004)**	0.45 (NS)	**0.64 (*****p*** **=** **0.01)**	**0.55 (*****p*** **=** **0.035)**	0.41 (NS)	0.22 (NS)
**PCA-Q**												
Visual complaint (/15)	−0.02 (NS)	0.41 (NS)	−0.02 (NS)	−0.13 (NS)	0.35 (NS)	−0.37 (NS)	−0.33 (NS)	−0.33 (NS)	−0.45 (NS)	0.42 (NS)	−0.51 (NS)	−0.02 (NS)
Gestural complaint (/9)	0.03 (NS)	0.31 (NS)	0.15 (NS)	−0.07 (NS)	0.20 (NS)	−0.01 (NS)	−0.27 (NS)	−0.29 (NS)	−0.38 (NS)	0.22 (NS)	−0.27 (NS)	0.03 (NS)

*Spearman coefficient correlations are reported in the table and significant p-values are specified in brackets (the statistical threshold is p < 0.05). Significant results, not corrected for multiple correlations, are reported in bold. NS, not significant*.

Regarding gestural functions, gestural performance was positively correlated to [^18^F]FDG uptake in the left supramarginal cortex and cerebellum (vermis excluded) using the voxel-wise analysis (see Table 2 and Figure 4) and uptake in the left lateral parietal and the bilateral medial parietal cortex using the ROI approach (see Table 3).

Visual complaint was negatively correlated to [^18^F]FDG uptake in the right cerebellum (vermis excluded) (x: 36, y:−70, z: −24; k = 155 voxels; T = 5.25) and positively correlated to [^18^F]FDG uptake in the left planum polare of the superior temporal gyrus (x: −42, y: −22, z: −4; k = 296 voxels, T = 6.81). No significant correlation was established with the ROI approach. Similarly, no correlation was found between gestural complaint and metabolism, regardless of approach.

## Discussion

The main aim of this study was to explore complaint, cognitive deficits and neuronal substrates in PCA patients. We found that the greater the impairment in terms of visual and gestural performance, the greater the hypometabolism in the temporo-parieto-occipital regions of the brain. However, no correlation between complaint and performance was established for visual and gestural domains.

Consistent with the previously described metabolic pattern of PCA (12, 13, 31), major hypometabolism was observed in the temporo-parieto-occipital cortex in patients compared to healthy subjects. “Occipital tunnel sign,” characterized by a relatively preserved metabolism in the medial occipital in comparison to the lateral occipital cortex was also found in our patients (32). This specific pattern is notably found in PCA and Dementia with Lewy Bodies (LDB) and could be useful to distinguish them from Alzheimer's disease (32). Metabolic hemispheric asymmetry is also found in PCA contrary to LDB (12, 33). In agreement with previous studies (14, 28), we found metabolic hemispheric asymmetry with right predominance in parietal and occipital cortices in PCA patients. Nestor et al. (14) postulated that hypometabolism predominance for the right or left hemisphere could be attributed to the choice of PCA patient recruitment (14). Left-sided hypometabolism is associated with additional deficits such as aphasia whereas major right hypometabolism could be indicative of a “purer” form of the PCA syndrome. In line with this hypothesis, Magnin et al. (34), explored logopenic syndrome in PCA (34). They reported no metabolic predominance of the left or right hemisphere in PCA patients presenting language disorders. However, patients who did not present logopenic syndrome displayed isolated right parieto-temporo-occipital hypoperfusion. In our study, the right hemisphere asymmetry observed may be the marker of a typical form of PCA but specific language assessments would have been helpful to confirm this hypothesis.

Based on the clinical classification revised by Crutch et al. (8), all our patients are considered as pure PCA with 12 patients defined as PCA-AD. Three patients did not present biomarkers consistent with AD pathology and may correspond to other pathophysiologies such as PCA-LBD or PCA-CBD (Cortico Basal Degenerarion) (8). Some authors have investigated amyloid and tau protein distribution using imaging in PCA patients. Amyloid was distributed diffusely throughout the neocortex in PCA patients (35) and did not differ from typical AD patients (36). However, localization of tau ligand retention seems to better correlate with hypometabolism patterns. Ossenkoppele et al. reported greater hypometabolism and greater aggregation of tau in PCA patients, as measured by [^18^F]AV1451 PET. Both are correlated and a strong overlap is reported in the right hemisphere and in the occipito-parietal cortex in particular (28). Other studies also reported an alteration of white matter in this pathology (37, 38). Cerami et al. even suggested that these alterations leading to a deafferentation could be at the origin of hypometabolism in PCA patients and therefore explain why the pattern of hypometabolism is more extensive than that of atrophy. Deafferentation processes within the occipital-parietal-frontal network may also explain visuospatial and visuoperceptive impairments (37). In our study, visual spatial deficits were not only associated with hypometabolism in the parietal cortex (bilateral superior supramarginal) but also in the occipital cortex. On the other hand, for perceptive deficits, a voxel-wise approach also suggested an association with decreased metabolism in the right inferior temporal gyrus in addition to superior parietal hypometabolism.

An alteration in temporal functioning in relation to visual perceptive tasks (ventral pathway), and in parietal functioning in relation to visuospatial tasks (dorsal pathway), have already been reported in the literature (11, 14). In our study, metabolism in the right superior parietal cortex was correlated with both perceptive and spatial visual modalities, and is highlighted via two different approaches (i.e., voxel-wise and ROI correlations). The constant involvement of this parietal cortex, already reported (13, 39), suggests a primary alteration in the parietal area during the initial stages of the disease, tending to extend to more temporal regions at later stages (39). Two recent longitudinal studies confirm these later changes with atrophy that tends to increase in superior temporal and middle frontal gyrus during the course of the disease (40, 41). In terms of gestural functions, performance was linked to metabolism in the left supramarginal gyrus. This result is consistent with the literature and suggests that this region is involved in the perception of space and location of limbs (42).

Croisile and Mollion reported a PCA patient's complaint comparable to ours (PCA-Q mean = 18.5/32 in their study vs. PCA-Q mean = 17.4 in our sample) with a minimum complaint score of 9/32 whereas their AD patients and healthy controls did not report any difficulties with maximum complaint scores of 8/32 and 6/32, respectively (PCA-Q mean AD patients: 2.7; mean Healthy Controls: 0.97) (6). However, despite this complaint and the presence of poor visual and gestural performance, we did not find any direct link between the two. One possible hypothesis to explain the absence of correlation between them is the lack of awareness of self-cognitive impairment in the patients enrolled in our study. This phenomenon has already been described in typical Alzheimer's disease (43) with the implication of temporal (44), frontal (45), or cingulate cortex functioning (46). In PCA syndrome, patients are described as being aware of their visual and gestural impairment. This continuing insight into PCA patients was initially reported by Benson et al. (1) with 5 clinical cases (1). Since then, an examination of the literature has revealed that insight is preserved in PCA patients but, in most cases, the principle study by Benson et al. (1), is mostly cited (5, 39) and never really studied. Tang-Wai et al. reported the lack of insight in 2 out of 40 PCA patients in 2004 (2). In 2012, Everhart et al. reported the PCA case of a 56-year-old woman who initially presented anxiety and panic-like symptoms but no insight into the severity of the cognitive impairment (7). Some studies report that unawareness of impairments is associated with increased apathy but lower depression in AD patients whereas PCA patients seem conversely present preserved insight with less apathy (47) but higher depression than AD patients [Mendez et al. (48), see Montembeault et al. (49) for review]. In our sample, we observed patients presenting with the same level of difficulty but a different level of complaint. For instance, looking at the cognitive profiles of our patients 1 and 5 (see Table 1), we observed that global cognitive assessment (MMSE) and visual impairment were comparable between the two, but their respective level of visual complaint differed (scores of 6/15 and 14/15 for patient 1 and 5, respectively). The misunderstanding of these difficulties might account for the lack of correlation between complaint and performance. As reported by Everhart et al. patients may be aware of their difficulties but are not able to correctly evaluate them or assess their severity (6, 7). This misinterpretation of difficulties in PCA patients may also account for the delayed diagnosis. In future studies, the PCA-Q could also be completed by the caregiver. The absence of correlation could also be attributed to the lack of statistic power. Given the relative rare nature of this condition, only 15 patients were enrolled in the study.

Finally, we sought to explore the relationship between metabolism and visual and gestural complaint. There have been reports of left superior temporal gyrus involvement in auditory short-term memory (50) and both the perception and production of speech (51). Significant impairment of visual functions could lead to the establishment of compensatory mechanisms in these PCA patients. However, the level of complaint registered may be influenced in some patients by the difficulty in characterizing the impairment or gauging its severity, and thus does not clearly reflect the actual symptoms. The relationship between visual complaint and cerebral metabolism is likely to reflect more complex mechanisms that cannot be deciphered from the present data.

## Conclusion

We explored the relationship between complaint, cognitive performance, and neuronal substrates in PCA patients. Despite the presence of visual and gestural complaints and visual and gestural impairments, no relationship was found between complaint and performance. We therefore believe that the misinterpretation of difficulties is a potential cause of delayed diagnosis. The ability to diagnose PCA therefore remains a challenge for clinicians as novel disease-modifying therapies dedicated to the initial stages of AD are being developed. PCA patients must be diagnosed as early as possible in order to benefit from this future treatment.

## Data Availability Statement

The datasets generated for this study are available on request to the corresponding author.

## Ethics Statement

The studies involving human participants were reviewed and approved by Comité de Protection des Personnes Sud-Ouest et Outre-Mer I. The patients/participants provided their written informed consent to participate in this study.

## Author Contributions

LG analyzed, interpreted the data, and drafted the manuscript for intellectual content. CC, PPa, and BP had a major role in the acquisition of data. LS-A revised the manuscript for intellectual content. PPé and JP conceptualized the study and revised the manuscript for intellectual content.

### Conflict of Interest

The authors declare that the research was conducted in the absence of any commercial or financial relationships that could be construed as a potential conflict of interest.
