# Supplementary material for: Posterior Cortical Atrophy: Does Complaint Match the Impairment? A Neuropsychological and FDG-PET Study
Source: Front Neurol. 2019 Sep 20;10:1010. doi: 10.3389/fneur.2019.01010 (PMC6764288; doi:10.3389/fneur.2019.01010)
Supplement: Supplementary file 1 [file Data_Sheet_1.docx]

**Supplementary Materials**

Supplementary, Figure 1.A: French version of PCA questionnaire

Supplementary, Figure 1.B: English version of PCA questionnaire

Supplementary, Appendix A.1: Details on the composition of visual and gestural complaint sub-scores

Supplementary, Appendix A.2: Description of complete visual and gestural assessment

Supplementary, Table 1: Visual and gestural performance for each PCA patient

Supplementary, Table 2: Combined ROIs from Desikan-Killiany-Tourville atlas

Supplementary, Table 3: Cognitive performance for each PCA patient

Supplementary, Table 4: FDG uptake and asymmetry index in regions of interest

| **Score Total du Q-ACP (nombre de oui) = / 32** | | |
| --- | --- | --- |
| Secteurs | Symptômes à rechercher | Réponses  Oui/Non/Na |
| Vision | 1 - Ne pas voir un objet devant soi |  |
|  | 2 - Paradoxalement, mieux repérer les objets éloignés que les objets proches |  |
|  | 3 - Voir flou |  |
|  | 4 - Se tromper d’ustensiles ou d’outils, confondre les objets entre eux |  |
|  | 5 - Ne plus reconnaitre certains visages |  |
| Regard | 6 – Avoir un regard mal dirigé, dans une mauvaise direction |  |
|  | 7 - Regard vague ou perdu |  |
| Lieux | 8 - Se perdre dans les lieux familiers (à pied, en voiture, en transports en communs) |  |
|  | 9 - Se tromper de sortie (de pièce, de lieu, de parking...) |  |
| Utilisation d’objets Saisie | 10 - Mal utiliser outils ou ustensiles (télécommande, électroménager, machines...) |  |
|  | 11 - Mal disposer verres, couverts et assiettes, casser beaucoup d’objets |  |
|  | 12 - Ne pls savoir disposer ses doigts sur les touches du téléphone |  |
| Saisie | 13 - Rater un objet à saisir ou une main à serrer |  |
|  | 14 - Tâtonner pour trouver ou prendre les objets |  |
| Fauteuils, chaises, lit | 15 - S’asseoir à l’envers ou de travers, rater le fauteuil, s’asseoir entre deux chaises, se coucher en travers du lit ou à l’envers |  |
| Portes | 16 - Ne pas trouver les poignées ou les serrures, lever la poignée au lieu de l’abaisser |  |
|  | 17 - Heurter les portes, les chambranles, les meubles |  |
| Voiture | 18 - Ne pas voir les trottoirs ou une voiture devant soi, perdre sa voiture sur le parking, se tromper de voiture ou ne pas la voir |  |
|  | 19 - En cas de conduite automobile : souvent rayer sa voiture ou celles des autres, rater les créneaux, mal évaluer les distances, rouler a` gauche ou au milieu de la rue |  |
|  | 20 - Se tromper de côté ou de portière de voiture |  |
|  | 21 - Chercher à tâtons la poignée de la portière |  |
|  | 22 - S’assoir à l’envers ou de travers, voire sur le fond |  |
|  | 23 - Ne plus savoir mettre sa ceinture de sécurité |  |
| Vêtements | 24 - Difficultés fréquentes d’habillage : se tromper de manche, avoir du mal à enfiler un pull ou une chemise, commettre des erreurs de boutonnage |  |
| Chaussures | 25 - Se tromper de chaussure ou de pied, mettre deux chaussures différentes |  |
| Lecture | 26 - Avoir l’impression que les lettres sont floues, se brouillent ou disparaissent lorsqu’on les fixe longuement |  |
|  | 27 - Se tromper de ligne lors du passage a` la ligne suivante |  |
|  | 28 - Lire plus facilement les petites lettres que les grosses lettres |  |
| Ecriture | 29 - Mal réaliser lettres et chiffres |  |
|  | 30 - Ne plus savoir signer |  |
|  | 31 - Espacer les lettres, agrandir les marges, oublier barres ou accents, disperser les mots, onduler les lignes |  |
|  | 32 - Faire des fautes d’orthographe |  |

Supplementary material, Figure 1.A: French version of PCA-questionnaire (1)
The visual complaint sub-score (/15) and the gestural complaint sub-score (/9) were calculated using the questions highlighted in orange and green, respectively.

| **Total Q-ACP Score (number of positive answers) = / 32** | | |
| --- | --- | --- |
| Sectors | Symptoms to test for | Answers  Yes/No/NA |
| Vision | 1 – Cannot see an object in front of you |  |
|  | 2 - Paradoxically, distant objects are more discernible than those close to you |  |
|  | 3 – Blurred vision |  |
|  | 4 – Confusion of utensils/tools, object confusion |  |
|  | 5 – You no longer recognise certain faces |  |
| Look | 6 – Poorly directed look, in the wrong direction |  |
|  | 7 – Vague or lost look |  |
| Location | 8 – Lose yourself in familiar locations (on foot, by car, on public transport) |  |
|  | 9 - Take the wrong exit (room, location, car park, etc.) |  |
| Use of objects  Grasp | 10 – Incorrect use of tools or utensils (remote control, electrical appliances, machines, etc.) |  |
|  | 11 – Poor handling of glasses, cutlery and plates; you break many objects |  |
|  | 12 – You no longer know how to position your fingers on the telephone keypad |  |
| Grasp | 13 – Miss an object to be picked up or a hand to be shaken |  |
|  | 14 – Fumble around to find or grasp objects |  |
| Armchairs, chairs, bed | 15 – Sit at the opposite end or across, miss the armchair, sit down between two chairs, lie across the bed or upside down |  |
| Doors | 16 – Unable to find handles or locks, lift the handle instead of lowering it |  |
|  | 17 – Bump into doors, door frames and furniture |  |
| Car | 18 – Do not see pavements or a car in front of you, lose your car in the car-park, go to the wrong car or do not see the car |  |
|  | 19 – When driving: often scratch your car or cars of other people, miss slots, misjudge distances, drive to the right or in the middle of the road |  |
|  | 20 – Go to the wrong side or car door |  |
|  | 21 – Fumble for the door handle |  |
|  | 22 –Sit upside down or across, even at the bottom |  |
|  | 23 – No longer knows how to put on a safety belt |  |
| Clothing | 24 – Frequent problems dressing: miss the sleeve, find it difficult to put on a jumper or shirt, do buttons up incorrectly |  |
| Shoes | 25 – Mistake the shoe or foot, put on two different shoes |  |
| Reading | 26 – Have the impression that the letters are blurred, become scrambled or disappear when you look at them for a prolonged period |  |
|  | 27 – Miss a line when moving to the next line |  |
|  | 28 – Read small letters more easily than big letters |  |
| Writing | 29 – Poor composition of letters and figures |  |
|  | 30 – No longer knows how to sign |  |
|  | 31 – Leave spaces between letters, enlarge the margins, forget bars or accents, separate/scatter words, wavy lines |  |
|  | 32 – Make spelling mistakes |  |

Supplementary material, Figure 1.B: English version of PCA-questionnaire (1)
The visual complaint sub-score (/15) and gestural complaint sub-score (/9) were calculated using the questions highlighted in orange and green, respectively.

Supplementary material, Appendix A.1: Details on the composition of visual and gestural complaint sub-scores

The visual complaint sub-score (/15) was calculated using questions 1 to 7, 13, 14, 16, 18, 21 and 26 to 28. The gestural complaint sub-score (/9) was calculated using questions 10 to 12, 15, 20 and 22 to 25. Eight questions were not selected either because they were not applicable to all patients (e.g. questions 19) or because they were not consistent with one of the sub-scores (questions 8, 11, 17, 29, 30, 31 and 32).

Supplementary material, Appendix A.2: Description of complete visual and gestural assessment

PCA patients underwent a visual function assessment using the Visual Object and Space Perception battery (VOSP) (2,3) and the Visual Gnosies Assessment Protocol (PEGV) (4). Primary visual capacities were assessed using the *Shape Detection Screening* test of the VOSP battery. Visuoperceptive functions were assessed with the following 3 tests taken from the VOSP battery: *Incomplete Letters* test assessing the ability to identify shapes, *Silhouettes* test assessing the capacity to identify objects depicted from unusual perspectives and the *Object Decision* test assessing the capacity to select the silhouette drawing of a real object from three nonsense objects. The *Identical Figures* and *Entangled Figures* tests from the PEGV battery were also used to assess visuoperceptive functions and, more precisely, simultagnosia. Visuospatial functions were evaluated with the *Dot Counting* test to identify the number of stimuli presented in random array, *Position Discrimination* and *Number Location* tests to discriminate relative spatial position and the *Cube Analysis* test to assess the ability to interpret three-dimensional space represented in two dimensions. Gestural functions were also evaluated by Mahieux’s test (Mahieux-Laurent et al., 2009) with *symbolic gestures*, *pantomimes* and *Imitation of meaningless gestures.*

Patients who failed at the example phase or who could not complete the test due to considerable impairment were deemed untestable. Tests with more than 5 untestable patients (representing 1/3 of patients) were removed from the analyses. Therefore, the following tests were not included in the analyses: 5 visual tests [*number location (n=7)*, *cube analysis (n=8)*, *incomplete letters* (n=6), *identical figures* (n=6), *entangled figures* (n=12) and *imitation of meaningless gestures (n=8)]*.
The performance of each of the afore-mentioned tests are described in detail in the supplementary material, Table 1.

| Patient Number | 1 | 2 | 3 | 4 | 5 | 6 | 7 | 8 | 9 | 10 | 11 | 12 | 13 | 14 | 15 | PCA  Median [IQR] | N below 5^th^ %ile (%) |  |
| --- | --- | --- | --- | --- | --- | --- | --- | --- | --- | --- | --- | --- | --- | --- | --- | --- | --- | --- |
| **PCA-Q** |  |  |  |  |  |  |  |  |  |  |  |  |  |  |  |  |  |  |
| Total /32 | 15 | 10 | 25 | 26 | 27 | 8 | 18 | 17 | 19 | 12 | 10 | 27 | 9 | 14 | 19 | 17 [11] |  |  |
| Visual questions /15 | 6 | 6 | 10 | 11 | 14 | 4 | 7 | 6 | 11 | 6 | 6 | 12 | 7 | 5 | 9 | 7 [4.5] |  |  |
| Gestural questions /9 | 5 | 2 | 7 | 7 | 5 | 2 | 6 | 5 | 4 | 3 | 1 | 8 | 0 | 3 | 8 | 5 [4] |  |  |
| **Visuoperceptive Functions** | | | | | | | | | | | | | | | | | | |
| *VOSP Battery* | | | | | | | | | | | | | | | | | | |
| Incomplete Letters /20 | UT | 2 | 9 | UT | UT | 10 | 14 | 10 | 10 | 12 | UT | UT | 13 | 18 | UT | 10 [3] | 14 (93.3%) |  |
| *PEGV Battery* |  |  |  |  |  |  |  |  |  |  |  |  |  |  |  |  |  |  |
| Identical Figures | 2 | 2 | 8 | UT | UT | UT | 4 | UT | 2 | 4 | 2 | UT | 9 | 10 | UT | 4 [6] | 13 (86.7%) |  |
| Entangled Figures | UT | UT | 25 | UT | UT | UT | UT | UT | UT | UT | UT | UT | 29 | 34 | UT | 29 [4.5] | 14 (93.3%) |  |
| **Visuospatial Functions** | | | | | | | | | | | | | | | | | | |
| *VOSP Battery* |  |  |  |  |  |  |  |  |  |  |  |  |  |  |  |  |  |  |
| Number Location /10 | UT | 5 | 9 | UT | UT | UT | 6 | 2 | 9 | 4 | UT | UT | 10 | 8 | UT | 7 [4.3] | 11 (73.3%) |  |
| Cube Analysis /10 | UT | 0 | 6 | UT | UT | UT | UT | 2 | 2 | 3 | UT | UT | 8 | 9 | UT | 3 [5] | 13 (86.7%) |  |
| **Gestural Functions** |  |  |  |  |  |  |  |  |  |  |  |  |  |  |  |  |  |  |
| *Mahieux Tests* |  |  |  |  |  |  |  |  |  |  |  |  |  |  |  |  |  |  |
| Abstract gestures /8 | UT | 2 | 6 | UT | UT | UT | UT | 8 | 4 | 6 | UT | UT | 5 | 8 | UT | 6 [2.5] | 13 (86.7%) |  |

Supplementary material, Table 1: Visual and gestural performance for each PCA patient
Raw scores for each patient are presented, along with the median and interquartile group range. Number and percentage of PCA patients with scores below the 5^th^ percentile are reported when possible.

Abbreviations: PCA, Posterior Cortical atrophy; IQR, Inter-quartile Range; PCA-Q, Posterior Cortical Atrophy Questionnaire; VOSP, Visual Object and Space Perception Battery; PEGV, Visual Gnosies Assessment Protocol; UT, Untestable

| Combined Region | Atlas Labels | Atlas Labels Names |
| --- | --- | --- |
| Frontal | 1003, 1012, 1014, 1018, 1019, 1020, 1027, 1028, 1032,  2003, 2012, 2014, 2018, 2019, 2020, 2027, 2028, 2032 | Left caudal middle frontal cortex, left lateral orbitofrontal cortex, left medialorbitofrontal cortex, left parsopercularis cortex, left parsorbitalis cortex, left parstriangularis cortex, left rostralmiddlefrontal cortex, left superiorfrontal cortex, left frontalpole cortex,  Right caudalmiddlefrontal cortex, right lateralorbitofrontal cortex, right medialorbitofrontal cortex, right parsopercularis cortex, right parsorbitalis cortex, right parstriangularis cortex, right rostralmiddlefrontal cortex, right superiorfrontal cortex, right frontalpole cortex |
| Lateral Temporal | 1001, 1009, 1015, 1030, 1034,  2001, 2009, 2015, 2030, 2034 | Left bankssts cortex, left inferior temporal cortex, left middle temporal cortex, left superior temporal cortex, left transverse temporal cortex,  Right bankssts cortex, right inferior temporal cortex, right middle temporal cortex, right superior temporal cortex, right transverse temporal |
| Medial Temporal | 17, 53,  1006, 1016,  2006, 2016 | Left Hippocampus, Right Hippocampus,  Left entorhinal cortex, left parahippocampal cortex,  Right entorhinal cortex, right parahippocampal |
| Lateral Parietal | 1008, 1029, 1031,  2008, 2029, 2031 | Left inferior parietal cortex, left superior parietal cortex, left supramarginal cortex,  Right inferior parietal cortex, right superior parietal cortex, right supramarginal |
| Medial Parietal | 1010, 1025,  2010, 2025 | Left isthmuscingulate cortex, left precuneus cortex,  Right isthmuscingulate cortex, right precuneus |
| Occipital | 1005, 1011, 1013, 1021,  2005, 2011, 2013, 2021 | Left cuneus cortex, left lateral occipital cortex, left lingual cortex, left pericalcarine cortex,  Right cuneus cortex, right lateral occipital cortex, right lingual cortex, right pericalcarine |

Supplementary material, Table 2: Combined ROIs from Desikan-Killiany-Tourville atlas

These ROIs are defined by Ossenkoppele et al. (2016), from Desikan-Killiany-Tourville atlas

| Patient Number | 1 | 2 | 3 | 4 | 5 | 6 | 7 | 8 | 9 | 10 | 11 | 12 | 13 | 14 | 15 | PCA Median [IQR] | N below 5^th^ %ile (%) |
| --- | --- | --- | --- | --- | --- | --- | --- | --- | --- | --- | --- | --- | --- | --- | --- | --- | --- |
| **General Functions** | | | | | | | | | | | | | | | | | |
| MMSE /30 | 17 | 20 | 24 | 9 | 15 | 14 | 28 | 22 | 25 | 20 | 15 | 15 | 23 | 25 | 12 | 20 [8.5] |  |
| FCSRT (Sum of free recalls, /48) | UT | 19 | 16 | UT | UT | UT | 26 | 15 | 21 | UT | 15 | UT | 26 | 35 | UT | 20 [10.3] |  |
| FCSRT (Sum of free & cued recalls, /48) | UT | 45 | 47 | UT | UT | UT | 46 | 36 | 38 | UT | 34 | UT | 43 | 45 | UT | 44 [7.75] |  |
| Digit Span (Forward) | 5 | 4 | 6 | 4 | 5 | 4 | 6 | 5 | 5 | 5 | 4 | 5 | 6 | 5 | 4 | 5 [1] |  |
| Digit Span (Backward) | 2 | 2 | 4 | 2 | 3 | 2 | 3 | 2 | 4 | 4 | UT | 3 | 5 | 4 | UT | 3 [2] |  |
| WAIS IV, Similarities (/36) | 8 | 12 | 20 | 14 | UT | 15 | 19 | 16 | 23 | 16 | 8 | 19 | 20 | 27 | UT | 17.5 [5.5] | 4 (26.6%) |
| **Language Functions** |  |  |  |  |  |  |  |  |  |  |  |  |  |  |  |  |  |
| “P” Fluency | 12 | 14 | 13 | 7 | 23 | 8 | 27 | 9 | 27 | 23 | 4 | 21 | 21 | 17 | 3 | 14 [13.5] | 5 (33.3%) |
| “Animals” Fluency | 16 | 21 | 9 | 8 | 11 | 10 | 38 | 11 | 22 | 17 | 14 | 8 | 12 | 23 | 10 | 12 [9] | 10 (66.6%) |
| Reading /24 | 3 | 21 | 23 | UT | UT | 9 | 21 | 23 | 19 | 22 | 11 | UT | 20 | 24 | 19 | 20.5 [5.3] |  |
| Dictation /13 | 4 | 3 | 10 | UT | 1 | 3 | 9 | 10 | 9 | 6 | 0 | 0 | 5 | 11 | 1 | 4.5 [7.5] |  |

Supplementary material, Table 3: Cognitive performance for each PCA patient
Raw scores for each patient are presented, along with the median and interquartile group range. The number and percentage of PCA patients with scores below the 5^th^ percentile are reported when possible.
Abbreviations: PCA, Posterior Cortical atrophy; IQR, Inter-quartile Range; MMSE, Mini Mental Scale Examination; FCSRT, Free and Cued Selective Reminding Test; WAIS, Wechsler Adult Intelligence Scale; UT, Untestable

|  | | Healthy Controls | | PCA Patients | |
| --- | --- | --- | --- | --- | --- |
|  |  | SUVR (median) | AI (%) | SUVR (median) | AI (%) |
| Frontal Cortex | Left | 1.03 | -1.46 | 0.79 | 1.63 |
|  | Right | 1.06 |  | 0.76 |  |
| Lateral Temporal Cortex | Left | 1.00 | -1.3 | 0.57 | 17.55 |
|  | Right | 1.02 |  | 0.52 |  |
| Medial Temporal Cortex | Left | 0.72 | 4.62 | 0.56 | 11.31 |
|  | Right | 0.69 |  | 0.51 |  |
| Lateral Parietal Cortex | Left | 1.04 | 0.46 | 0.48 | 22.65 |
|  | Right | 1.04 |  | 0.38 |  |
| Medial Parietal Cortex | Left | 1.26 | 7.26 | 0.68 | 16.76 |
|  | Right | 1.18 |  | 0.52 |  |
| Occipital Cortex | Left | 1.19 | 0.72 | 0.59 | 34.93 |
|  | Right | 1.18 |  | 0.45 |  |

Supplementary, Table 4: FDG uptake and hemispheric asymmetry index in regions of interest

A hemispheric asymmetry index (AI) was calculated for each region of interest using the formula AI [%] = - 200 x (R - L)/(R + L).

Abbreviations: SUVR, Standardized Uptake Value Ratio; AI cortex, Asymmetry Index

**REFERENCES:**

1. Croisile B, Mollion H. Q-ACP : un questionnaire d’évaluation des plaintes visuelles et gestuelles des patients ayant une atrophie corticale postérieure. *Rev Neurol (Paris)* (2011) **167**:485–494. doi:10.1016/J.NEUROL.2010.11.003

2. Rapport LJ, Millis SR, Bonello PJ. Validation of the Warrington Theory of Visual Processing and the Visual Object and Space Perception Battery*. *J Clin Exp Neuropsychol* (2002) **9800**:1380–3395.

3. Bonello P, Rapport L, Millis S. Psychometric properties of the visual object and space perception battery in normal older adults. *Clin Neuropsychol* (1997) **11**:436–442. doi:10.1080/13854049708400475

4. Agniel A, Joanette Y, Doyon B, Duchein C. *P.E.G.V.: protocole d’évaluation des gnosies visuelles.* OrthoEditi. Montréal–Toulouse (1987).

5. Mahieux-Laurent F, Fabre C, Galbrun E, Dubrulle A, Moroni C. Validation of a brief screening scale evaluating praxic abilities for use in memory clinics. Evaluation in 419 controls, 127 mild cognitive impairment and 320 demented patients. *Rev Neurol (Paris)* (2009) **165**:560–567.
